# A Novel Dual-Color Reporter for Identifying Insulin-Producing Beta- Cells and Classifying Heterogeneity of Insulinoma Cell Lines

**DOI:** 10.1371/journal.pone.0035521

**Published:** 2012-04-18

**Authors:** Nan Sook Lee, Joyce G. Rohan, Madison Zitting, Sonia Kamath, Andrew Weitz, Arnold Sipos, Paul M. Salvaterra, Kouichi Hasegawa, Martin Pera, Robert H. Chow

**Affiliations:** 1 Department of Physiology & Biophysics and Zilkha Neurogenetics Institute, University of Southern California, Los Angeles, California, United States of America; 2 Department of Biomedical Engineering, University of Southern California, Los Angeles, California, United States of America; 3 School of Medicine, University of Southern California, Los Angeles, California, United States of America; 4 Division of Neuroscience, Beckman research Institute of the City of Hope, Duarte, California, United States of America; 5 Eli and Edythe Broad Center for Regenerative Medicine and Stem Cell Research, University of Southern California, Los Angeles, California, United States of America; University of British Columbia, Canada

## Abstract

Many research studies use immortalized cell lines as surrogates for primary beta- cells. We describe the production and use of a novel “indirect" dual-fluorescent reporter system that leads to mutually exclusive expression of EGFP in insulin-producing (INS^+^) beta-cells or mCherry in non-beta-cells. Our system uses the human insulin promoter to initiate a Cre-mediated shift in reporter color within a single transgene construct and is useful for FACS selection of cells from single cultures for further analysis. Application of our reporter to presumably clonal HIT-T15 insulinoma cells, as well as other presumably clonal lines, indicates that these cultures are in fact heterogeneous with respect to INS^+^ phenotype. Our strategy could be easily applied to other cell- or tissue-specific promoters. We anticipate its utility for FACS purification of INS^+^ and glucose-responsive beta-like-cells from primary human islet cell isolates or *in vitro* differentiated pluripotent stem cells.

## Introduction

Diabetes prevalence is increasing dramatically worldwide and severe co-morbidities persist, despite the availability of insulin treatment. Cell replacement strategies are thus being developed to treat this metabolic disease. A key treatment step will be production of sufficient quantities of fully functional pancreatic beta- or beta-like-cells suitable for replacing missing or defective beta-cells. This goal has stimulated renewed interest in understanding human islet cell biology. However, because of the difficulty and high cost associated with isolating human islets, most studies focus on *in vitro* characterization of immortalized human or animal cell lines as surrogates for primary beta-cells.

Rodent insulinoma cell lines derived from cancers arising after radiation treatment (rat RIN, INS-1, CRI-G1) or viral transformation (hamster HIT, βHC) have been especially useful models of beta-cell biology; at the time of their establishment, they exhibit high levels of insulin production and glucose responsiveness [1], [2], [3], [4]. However, both of these pancreatic beta-cell attributes are lost with time in culture and increased numbers of cell passages [5]. Unfortunately, the generation and characterization of human insulinoma or beta-cell-derived cell lines that preserve normal glucose responsiveness has not been reported. The hamster insulinoma cell line, HIT-T15, has been one of the most extensively studied beta-cell-like models. HIT-T15 cells exhibit glucose-stimulated insulin secretion and contain membrane-bound secretory granules [6], similar to those seen in normal islet beta-cells. HIT-T15 cells were originally produced by SV40 transformation of pancreatic beta-cells, followed by serial selection of clonal lines expressing the glucose-responsive phenotype [7].

In this study, we describe the development and application of a new dual-color fluorescent reporter system for identifying insulin-producing (INS^+^) beta- and non-insulin-producing (INS^-^) cells. Our reporter contains a single transgene with two expression cassettes. The first is a fragment of the human insulin gene promoter (phINS) that drives expression of Cre recombinase protein exclusively in INS^+^ cells. The second contains the CMV promoter and an mCherry coding region flanked with LoxP (L) sites, followed by an EGFP coding region. In cells with active insulin promoter activity, the Cre protein excises the mCherry coding region, and the cells exhibit green fluorescence. In cells with no insulin promoter activity, the mCherry coding region is not excised, so the cells exhibit red fluorescence. This new “indirect" reporter strategy uses mutually exclusive expression of green or red fluorescence to eliminate ambiguity observed when a human insulin promoter directly drives expression of EGFP in combination with a CMV-regulated mCherry. Distinguishing INS^+^ from INS^-^ cells with the “direct" strategy depends on identifying cells that are doubly fluorescent and often leads to ambiguous results—the relative levels of fluorescence for the two reporter colors can be highly variable (due to variability in the relative strength of the two promoters driving fluorescent protein expression, differences in the relative fluorescence intensities, and/or relative rate of degradation of the proteins). Our “indirect" dual-color system, in contrast, reports all cells that have been transduced or transfected, so efficiency of transduction/transfection is easily calculated.

Regardless of which fluorescent protein is expressed, expression is under control of the same CMV promoter. We thus observe only a single color for each phenotype that reports successful *Ins* gene expression by a discrete change in color from red to green. In addition, our approach results in separate colors arising from the same transfected cells using a single transgene construct, which is not possible with a single-color reporter.

We prepared HIT-T15 cells stably transfected with our new reporter construct. Contrary to expectations, we observed that these presumably clonal cells are in fact heterogeneous with respect to a variety of beta-cell-like biochemical and electrophysiological phenotypes. After FACS isolation of green fluorescent cells, we obtained a stable, homogeneous population of insulin-producing cells with beta-like phenotypes. Since our reporter system uses the human insulin promoter, we anticipate its utility to identify and FACS purify INS^+^ and glucose-responsive beta-like-cells from primary human islet cell isolates or *in vitro* differentiated human pluripotent stem cells.

## Results

### Specificity of the proximal 378-nt region in the human insulin promoter

**Figure 1:**
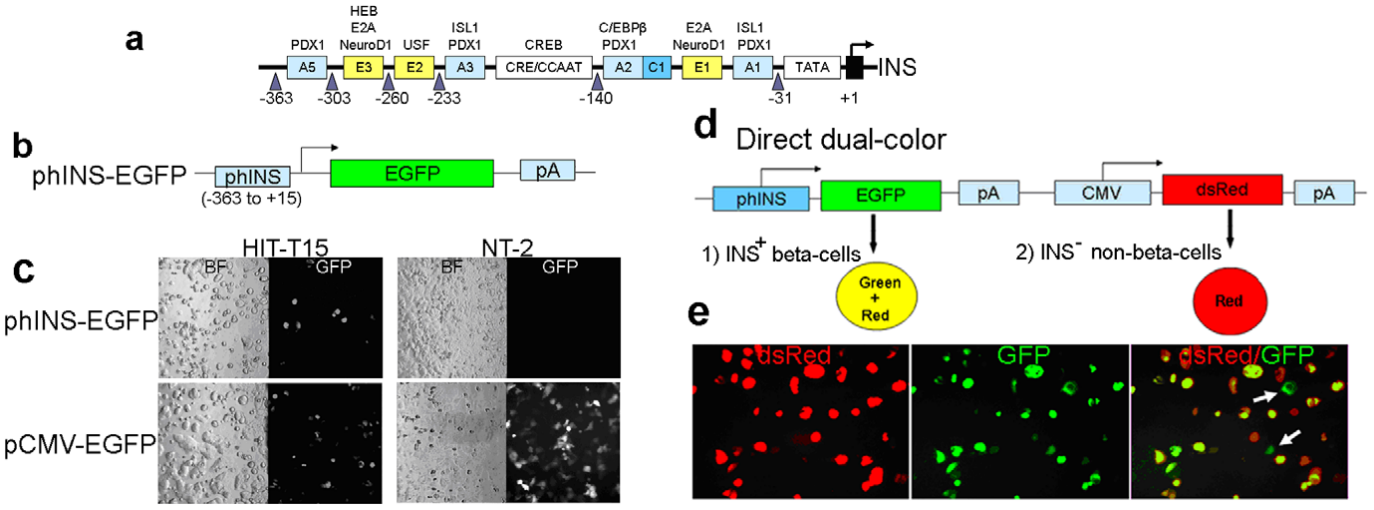
Specificity of the human insulin promoter (378-nt region) to identify insulin-producing cells and a direct dual-color reporter system. (a) Schematic picture of the proximal 378-nt region (−363 to +15) of the human insulin promoter containing several regulatory motifs for expression of the insulin gene. (b) The phINS-EGFP construct [6] used to express EGFP in insulin-producing cells. (c) phINS-EGFP was transfected into HIT-T15 or NT-2 cells. After 2 days, some HIT-T15 cells expressed EGFP, but NT-2 cells did not. When pCMV-EGFP was transfected, both cell lines expressed EGFP, suggesting the specificity of the 378-nt region to identify insulin-producing cells. (d) Schematic picture of a direct dual-color reporter that contains a human insulin promoter driving expression of EGFP and a CMV promoter driving expression of dsRed (phINS-EGFP-CMV-dsRed). (e) Stable transfection of phINS-EGFP-CMV-dsRed into HIT-T15. Many cells displayed red or yellow fluorescence, but a small population of cells unexpectedly appeared to express primarily green fluorescence (white arrows).

The complete human insulin promoter/enhancer is estimated to be ∼4 kb in length [8], [9]. The proximal 378-nt region (−363 to +15) is highly conserved between humans and rodents [10], [11]. It contains important regulatory motifs (Fig. 1a) previously used to make reporters for identifying INS^+^ cells [6], [12]. We first confirmed the utility of this region to report insulin production by transfecting a phINS-EGFP construct (Fig. 1b, [6]) into the HIT-T15 hamster insulinoma clonal cell line (insulin-producing) [7], as well as the NT-2 human embryonic carcinoma cell line (non-insulin-producing). As expected, only HIT-T15 cells exhibited green fluorescence (Fig. 1c). In contrast, using the ubiquitous CMV promoter instead of the insulin promoter to drive EGFP expression resulted in fluorescence of both cell types (Fig. 1c). We therefore concluded that this 378-nt promoter region is sufficient for marking insulin-producing cells, and we used this region to construct additional reporters.

### “Direct " dual-color reporter system

We modified phINS-EGFP by introducing a dsRed reporter cassette under control of the CMV promoter, reasoning that this could be used to control for transfection efficiency and to identify non-insulin producing cells. We expected INS^−^ cells to exhibit only red fluorescence, and INS^+^ cells to exhibit both red and green (yellow) fluorescence. Transient (data not shown) and stable transfection of phINS-EGFP-CMV-dsRed (Fig. 1d) into HIT-T15 cells resulted in cells that displayed red or yellow fluorescence (Fig. 1e). However, a small population of cells unexpectedly appeared to express primarily green fluorescence (Fig. 1e). We reasoned that this unexpected fluorescence was caused by unequal accumulation of fluorescent reporter protein in certain cells with different levels of expression [13] or unequal catabolism of the two fluorescent proteins [14]. Alternatively, transcriptional inactivation of promoters could have occurred during selection of stably transfected cells [15], [16], [17], [18]. Nevertheless, the presence of the green fluorescent cells complicated clear identification of cells with INS^+^ versus INS^−^ phenotypes, so we decided to try a new “indirect" approach.

### “Indirect" dual-color reporter system

Rather than use the insulin promoter to directly drive EGFP expression, we tested an alternate strategy in which the insulin promoter drives expression of Cre recombinase, leading to excision of an mCherry coding region located between CMV promoter and EGFP coding region. The mCherry coding region is followed by a stop codon; thus, the new design leads to mutually exclusive expression of either red or green fluorescent proteins.

**Figure 2:**
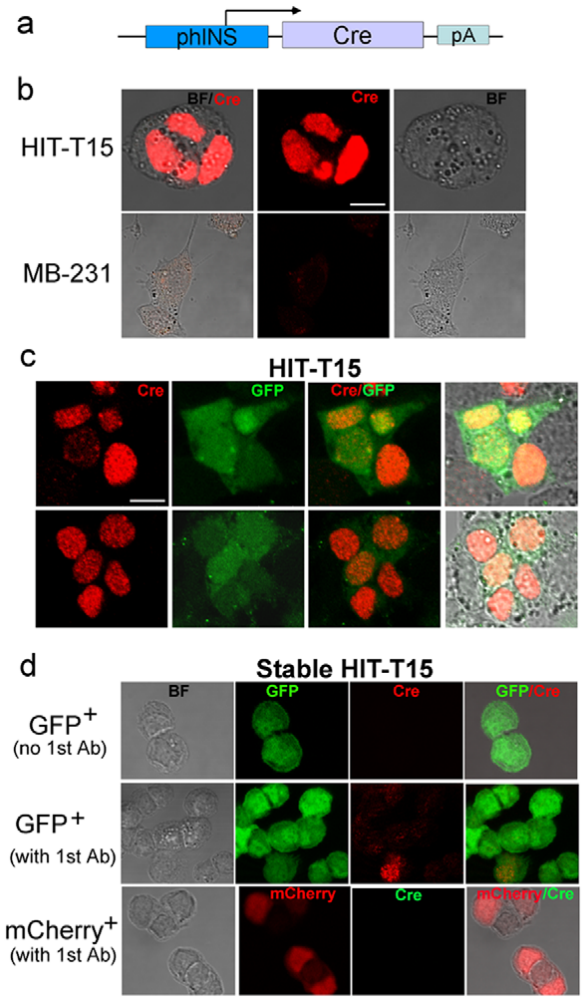
Expression of Cre recombinase from phINS-Cre plasmid. (a) Schematic picture of phINS-Cre construct. (b) Immunofluorescence analysis of HIT-T15 and MB-231 cells transiently transfected with phINS-Cre. The red color indicates Cre recombinase expression. Scale bar: 10 µm. (c) Immunostaining of HIT-T15 cells transiently co-transfected with phINS-Cre and pEGFP-N1. The red color indicates Cre recombinase expression (Cy-3), and the green is a marker for co-transfection. (d) Immunostaining of stable HIT-T15 FACS-sorted GFP^+^ and mCherry^+^ cells with and without the primary anti-Cre antibody. The green colors in the top and middle panels were visualized as EGFP expression, and the red (Cy-3) as Cre recombinase expression. The stable FACS-sorted mCherry^+^ cells in the bottom panels did not express Cre recombinase (no green, Cy-2).

To confirm that insulin promoter activity led to expression of Cre recombinase, we constructed a plasmid containing the Cre gene under the human insulin promoter (phINS-Cre-pA; Fig. 2a). This plasmid was transiently transfected into HIT-T15 cells, as well as MDA-MB-231 cells (non-insulin-producing negative controls). Roughly 70% of HIT-T15 cells became Cre^+^ (red), while MB-231 cells did not show Cre expression (Fig. 2b). When transiently transfected with pEGFP-N1, ∼70% of cells showed EGFP expression for both cell lines, indicating similar transfection efficiencies (Fig. S1). When HIT-T15 cells were transiently co-transfected with phINS-Cre and pEGFP-N1 (Fig. 2c), ∼70% of HIT-T15 cells were Cre^+^ (red), but not all red cells were green. This suggests that not all cells were transfected with both plasmids and motivated us to use a single construct containing a combination of both cassettes.

**Figure 3:**
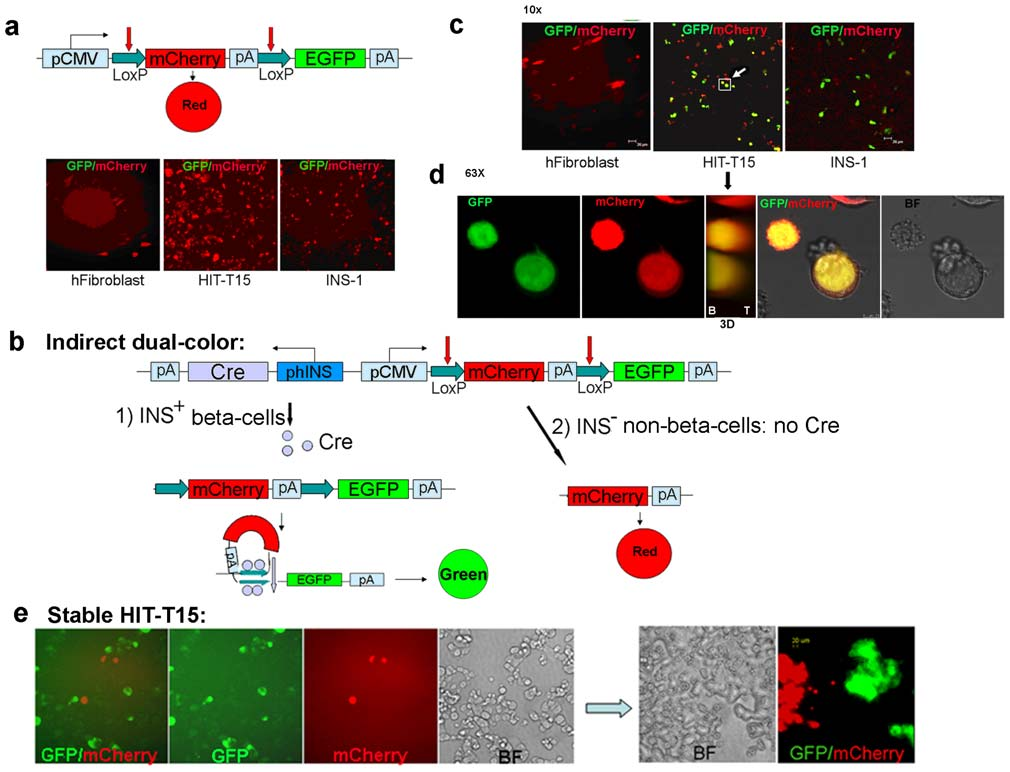
“Indirect" dual-color design of the reporter construct. (a) Transfection of cells with CMV-L-mCherry-L-EGFP. Without Cre recombinase in the construct, all cells exhibited red fluorescence. (b) Schematic and anticipated behavior of pA-Cre-phIns-CMV-L-mCherry-L-EGFP. INS^+^ beta-cells transfected by this plasmid should activate Cre recombinase expression and excise the mCherry reporter by Cre-LoxP recombination, thus converting red fluorescence to green. In contrast, transfected INS^−^ non-beta-cells should only exhibit red fluorescence. (c) After transfection of cells with pA-Cre-phIns-CMV-L-mCherry-L-EGFP, HIT-T15 and INS-1 expressed EGFP, indicating that mCherry was excised by Cre recombinase expressed under the human insulin promoter. This demonstrates the reporter's specificity to produce EGFP in only INS^+^ cells. (d) Highly magnified (63×) projection images of two yellow cells marked as the white square with a white arrow in Fig. 2c from Z-stacks (10×), illustrating both red and green fluorescence. In the middle panel, the 3D picture shows co-localization of both colors (B, bottom; T, top). (e) Stable HIT-T15 cell population containing the “indirect" dual-color construct (Neo^r^). ∼70% of the stably transfected cells were green, indicating mCherry excision. Another ∼10% were red, indicating the absence of mCherry excision and suggesting that the insulin promoter was never activated. The remaining ∼20% of cells exhibited no fluorescence, suggesting transcriptional inactivation of the CMV promoter. The right panel depicts the cells after more than 6 days in culture, compared to cells at 1 day after splitting shown in the left panel. No yellow fluorescent cells were observed. These results suggest that at least two phenotypically heterogeneous cells arise in the HIT-T15 cell culture model.

To make the single construct, we first constructed a plasmid containing only the fluorescent reporters (pCMV-L-mCherry-L-EGFP) (Fig. 3a). This plasmid was transiently transfected into human fibroblasts (a non-insulin-producing negative control), as well as in HIT-T15 cells and the rat insulinoma cell line, INS-1 823/13 [19]. The latter two cell lines are INS^+^ but should not show any red-to-green color change in the absence of a source of Cre. As expected, all transfected cells showed only red fluorescence (Fig. 3a).

We next constructed [pA-Cre-phINS(reverse orientation)]-pCMV-L-mCherry-L-EGFP by inserting a phINS-Cre-pA cassette fragment (Fig. 2a) upstream of pCMV-L-mCherry-L-EGFP in reverse orientation (Fig. 3b). Transfected INS^+^ cells should activate Cre recombinase expression and excise the mCherry reporter by Cre-LoxP recombination, thus converting red fluorescence to green. In contrast, transfected INS^–^ cells should only exhibit red fluorescence. We refer to this reporter as “indirect," since phINS does not directly drive EGFP expression, but rather changes the color of the reporter being expressed under control of the ubiquitous CMV promoter.

Transiently transfected fibroblasts showed only red fluorescence (Fig. 3c), indicating the absence of any Cre-mediated recombination. In contrast, transiently transfected HIT-T15 and INS-1 823/13 cells showed either red or green fluorescence, indicating Cre-mediated excision of the mCherry cassette in a subset of the transfected cells. We also observed a small number of HIT-T15 and INS-1 823/13 cells with yellow fluorescence (Fig. 3c), indicating expression of both red and green fluorescent proteins (Fig. 3c). We imaged the cells with confocal microscopy to confirm that the yellow cells showed both green and red fluorescence, rather than being two separate cells stacked atop one another (Fig. 3d, Fig. S2). The yellow fluorescence may be a technical artifact due to unequal copy numbers of our test plasmid in different cells [13] or unequal intracellular catabolism of red versus green fluorescent proteins [14]. We therefore produced stably transfected HIT-T15 cells using G418 selection for a neomycin resistance gene (Neo) incorporated in our plasmid to present 1–2 copies of the plasmid.

We expected all stably transfected cells to exhibit green fluorescence, since HIT-T15 is believed to be a clonal model of beta-like-cells and all should have activated the insulin promoter and expressed Cre [20]. However, we found that only ∼70% of the stably transfected cells were green, indicating mCherry excision (Fig. 3e, left panel). Another ∼10% were red, indicating the absence of mCherry excision and suggesting that the insulin promoter was never activated. The remaining ∼20% of cells exhibited no fluorescence, suggesting that transcriptional inactivation of the CMV promoter might have occurred during selection of stably transfected cells, although conflicting data about the activity of the CMV promoter in rodent undifferentiated embryonic stem cells (ESCs) have also been obtained [15], [16], [17], [18]. No yellow fluorescent cells were observed (Fig. 3e). These results suggest that at least two phenotypically heterogeneous cells arise in the HIT-T15 cell culture model. We could not select stably transfected INS-1 823/13 cells because they already contain a pCMV8/INS/IRES/Neo insertion [19].

**Figure 4:**
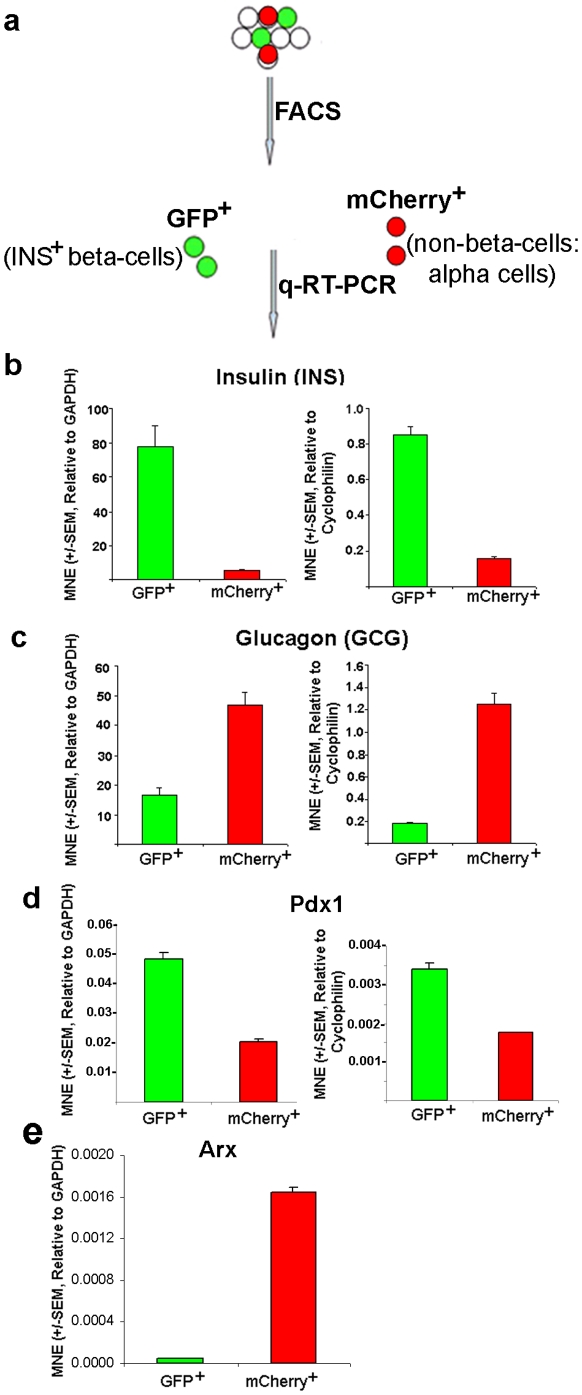
INS and GCG transcript expression in FACS-sorted GFP^+^ and mCherry^+^ cells using q-RT-PCR. (a) After FACS analysis in stable HIT-T15 cell population to select GFP^+^ or mCherry^+^ cells, q-RT-PCRs were performed in GFP^+^ and mCherry^+^ cells. (b, c) q-RT-PCR data confirmed that the green and red cells were insulin- and glucagon-producing, respectively. (d, e) The green and red cells were analyzed for expression levels of Pdx1 (d) and Arx (e) genes by q-RT-PCR. The expression levels of INS, GCG, Pdx1, and Arx transcripts were normalized to the housekeeping genes, GAPDH and/or Cyclophilin (“MNE"). The error bars represent standard error (SEM) of the mean (N = 3 for each experiment).

FACS sorting of the stably transfected HIT-T15 cells followed by q-RT-PCR analysis of each population directly confirmed their heterogeneity with respect to INS gene expression (Fig. 4). High levels of INS transcript were detected in the green population (Fig. 4b), while larger amounts of glucagon (GCG) were detected in the red population (Fig. 4c). Pdx-1 transcripts were expressed at higher levels in GFP^+^ cells relative to mCherry^+^ cells (Fig. 4d). The trends were similar, regardless of the reference gene (GAPDH vs. Cyclophilin). FACS-sorted stable GFP^+^ cells showed much weaker Cre recombinase (red, Cy-3) expression (Fig. 2d) (1–2 copies of the plasmid) relative to the transiently transfected cells (several copies) (Fig. 2b & c), confirming INS^+^ phenotype. In contrast, FACS-sorted mCherry^+^ cells did not express Cre recombinase (no green, Cy-2) (Fig. 2d, bottom panel), indicating INS^−^ phenotype. Furthermore, FACS-sorted GFP^+^ and mCherry^+^ cells grown separately in culture maintained their fluorescence throughout five subsequent passages, thus appearing to be stable. These data establish that the color shift from red to green using our reporter system faithfully indicates the status of INS gene expression. They also suggest that the original HIT-T15 population may have been heterogeneous with respect to INS^+^ phenotype (i.e., a population of GCG^+^ cells was also present), or perhaps cells change from INS^+^ to GCG^+^ in culture by overexpression of aristaless-related homeobox (Arx) (i.e., converting beta-cells into alpha-cells [21], [22]). Figure 4e supported the latter case, but we are further characterizing both cell populations by RNA sequencing analysis.

### Electrophysiological characteristics of FACS-sorted GFP^+^ and mCherry^+^ cells

**Figure 5:**
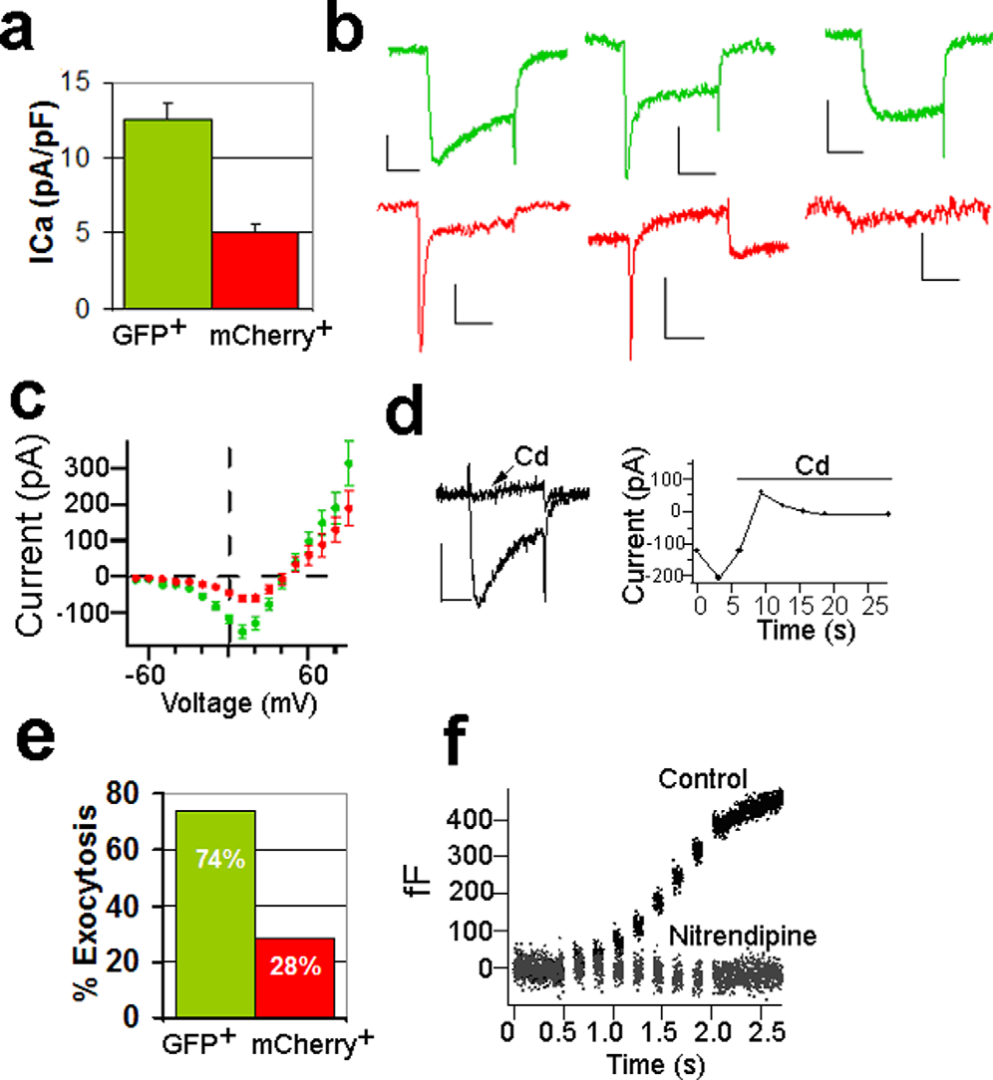
Electrophysiological differences between GFP^+^ and mCherry^+^ cells. (a) GFP^+^ cells exhibited significantly greater calcium currents compared to mCherry^+^ cells (Mann Whitney, P<0.0001, n = 19–22). (b) Example current traces from GFP^+^ cells (top panel) and mCherry^+^ cells (bottom panel). Scale bar: 100 pA, 10 ms. (c) Average current-voltage plot showing greater calcium influx in GFP^+^ cells (closed circles) compared to mCherry^+^ cells (open circles). (d) Cadmium (Cd^2+^, 30 mM) blocked inward current in a GFP^+^ cell. (e) Depolarizing trains of 8 pulses (100 ms, 0 mV) induced an increase in capacitance (corresponding to an increase in exocytosis) from most of GFP^+^ cells (21 out of 30). Only 7 out of 25 cells mCherry^+^ cells underwent an increase in capacitance when stimulated by 8-pulse depolarizing trains. (f) Capacitance recording from the same GFP^+^ cells with and without 20 µM Nitrendipine.

We further characterized the FACS-sorted INS-expressing green and non-INS-expressing red cells electrophysiologically. Using a standard whole-cell patch- clamp configuration, we found that the green cells exhibited more than a two-fold greater inward current than the red cells (Fig. 5 a–c; P<0.0001). The kinetics of the inward current were characteristic of Ca^2+^ channels, an idea further supported by the observation that addition of Cd^2+^ to the bath effectively blocked the current (Fig. 5d). We also found that a greater proportion of stimulated green cells underwent exocytosis compared to red cells [74% (21 out of 30) versus 28% (7 out of 25)], as inferred from changes in membrane capacitance following trains of eight depolarizing pulses (from −80 to 0 mV, 100 ms duration; Fig. 5e & f). Adding Cd^2+^ blocked changes in membrane capacitance, as would be expected for insulin secretion, which is known to be predominantly Ca^2+^-dependent (Fig. 5d).

The main voltage-gated Ca^2+^ channels controlling insulin secretion in beta-cells are L-type channels that can be blocked by nitrendipine [23]. Addition of 20 µM nitrendipine only partially blocked the Ca^2+^ current (∼50%) but almost totally eliminated the capacitance increase in green cells (Fig. 5f). This suggests that more than one type of Ca^2+^ channel is present, but that L-type Ca^2+^ channels gate the Ca^2+^ entry that is coupled to exocytosis. There was a greater variability in the amplitude of calcium currents and capacitance changes in red cells compared to green cells.

**Figure 6:**
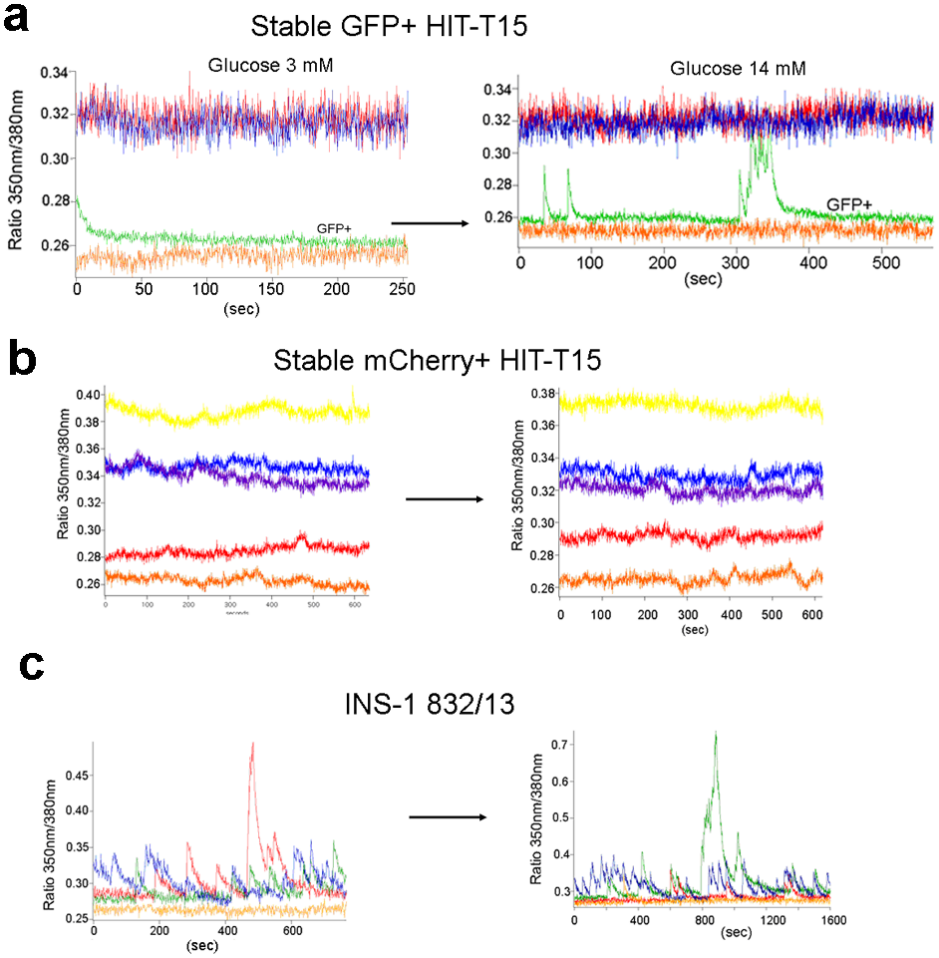
Intracellular Ca^2+^ concentration, [Ca^2+^]_i_ changes associated with glucose stimulation in different fluorescent cells. (a) Glucose responsiveness of GFP^+^ stable HIT-T15 cells. The green line represents a GFP^+^ cell in basal (3 mM, left panel) and stimulatory (14 mM, right panel) levels of glucose. The other lines (red, blue, and orange) represent non-fluorescent cells. (b) No glucose responsiveness of mCherry^+^ stable HIT-T15 cells was observed. (c) Fluorescence traces from INS-1 cells. Each color represents a different cell, indicating spontaneous Ca^2+^ oscillations arising from action potentials.

One characteristic of beta-cells is that intracellular Ca^2+^ levels ([Ca^2+^]_i_) rise in response to increased extracellular glucose [24], [25]. We used ratiometric imaging of the membrane-permeant calcium indicator dye, Fura2-AM (Fura2 acetoxymethyl ester [26]), in cells exposed to either basal (3 mM) or stimulatory (10–14 mM) levels of glucose. The fluorescence ratio (350 nm/380 nm) increased when a green cell was exposed to the stimulatory level of glucose (compare the green line in the left and right panels of Fig. 6a), indicating an increase in [Ca^2+^]_i_. The other colored lines in Figure 6a represent non-fluorescent cells, which do not exhibit a fluorometric ratio change under the same conditions. Ratiometric analysis of red cells did not show any change when shifted from the basal to the stimulatory glucose concentration (Fig. 6b). We also performed ratiometric imaging on INS-1 823/13 cells and observed increased [Ca^2+^]_i_ in both basal and stimulating glucose concentrations (Fig. 6c). INS-1 823/13 cells, which retain insulin over-expression under a ubiquitous promoter (in contrast to HIT-T15 cells), thus appear to have spontaneous [Ca^2+^]_i_ oscillations that are independent of glucose concentration.

Table 1 presents a summary of our phenotypic analysis of FACS-sorted stably transfected HIT-T15 cells. The results support our conclusion that our new “indirect" dual color reporter is an effective tool to distinguish a range of beta-cell-like phenotypes.

**Table 1:** Summary of beta-cell-like phenotypes in FACS-sorted HIT-T15 cells.

	Green Cells (GFP^+^)	Red Cells (mCherry^+^)
Ca^2+^ current density^a^	Larger	Smaller
Extent of exocytosis^b^	Larger	Smaller
Glucose-stimulated increase in [Ca^2+^]_i_	Yes	No
Insulin gene transcription	Yes (high level)	Yes (low level)
Glucagon gene transcription	Yes (low level)	Yes (High level)

a Ca^2+^ current density is measured in pA/pF (current divided by capacitance), a measure of the flow of calcium in the membrane area. GFP^+^ cells showed 12.6±1 pA/pF, while mCherry^+^ cells showed 5±0.61 pA/pF (p<0.0001).

b The percentage of cells exhibiting capacitance increase was 74% for GFP^+^ cells and 28% for mCherry^+^ cells.

## Discussion

In the present study, we describe the design and use of a new “indirect" fluorescent reporter system that drives mutually exclusive expression of either EGFP for INS^+^ cells or mCherry for INS^−^ cells. This new approach relies on a human insulin promoter-driven Cre-mediated shift in reporter color from red to green in single transgene construct. This results in several advantages over other approaches that use single- or dual-color reporters to distinguish cellular phenotypes.

A single-color reporter system with the insulin promoter driving expression of a fluorescent protein [27], [28], [29] serves to identify a subset of cells fulfilling two conditions: (1) successful transduction/transfection and (2) activation of the insulin promoter. However, this system does not allow for straightforward calculation of the percentage of transfected/transduced cells. Among those cells that are successfully transfected/transduced, it is not clear what fractions have activated the insulin promoter.

The “direct" dual-color reporter systems that have been described use the ubiquitous CMV or a cell-/tissue-specific promoter to drive one color and another cell-/tissue-specific reporter for the second color [30], [31], [32], [33], [34], [35]. Thus, the cells in which the cell-/tissue-specific promoter is activated should express two colors, while all other cells should express only one color. In principle, this system has the advantage compared to the single-color system of identifying all cells that have been successfully transfected or transduced, so the efficiency of transfection/transduction can be easily calculated. However, a problem with this system became evident when we employed this approach (Fig. 1d & e). For cells in which the tissue-specific promoter had been activated, the relative levels of fluorescence for the two reporter colors was highly variable, due to variability in the relative strength of the two promoters driving fluorescent protein expression, differences in the relative fluorescence intensities, and/or relative degradation rate of the proteins. In a small subset of cells, the only fluorescence seen was that expected to be due to the activation of the cell-/tissue-specific promoter. It is also possible that in these cells, the CMV promoter was inactivated [15], [16], [17], [18].

The “indirect" dual-color reporter system that we have introduced reports all cells that have been transduced/transfected (so efficiency of transduction/transfection is easily calculated), and it also leads to mutually exclusive marking of cells with one or the other fluorescent protein. Regardless of which fluorescent protein gets expressed, expression is under control of the same strong and ubiquitous CMV promoter. We thus observe only a single color for each phenotype that reports successful INS gene expression by a discrete change in color from red to green (Fig. 3e and Fig. 4b).

Another advantage of our indirect dual-color reporter relates to possible off-target effects of the introduced plasmid or viral vector. Introduction of plasmid or viral vectors into cells may alter cellular phenotype [36], [37], due to heterologous expression of non-native viral and inserted proteins. Controlling for such changes using a single-color reporter is not possible, since different cultures with control vectors are used to assess phenotypes [29]. In contrast, our approach results in separate colors arising from the same transfected cells using a single transgene construct. The color resulting from the Cre-mediated recombination event (i.e., removal of the mCherry cassette) thus allows GFP^+^ cells to serve as an appropriate control for non-insulin-positive cell phenotypes. A very similar dual-color system to ours was reported [38], but the investigators used two separate lentiviral constructs that could not eliminate the complexity of cell-to-cell transduction variability ([32], Fig. 2c). Nevertheless, our reporter may need to discern gradients of insulin promoter activity (red-to-green cells over time) in transiently transfected cells using real-time imaging.

Using our new reporter, we found that presumably clonal HIT-T15 cells [7], [20] were heterogeneous with respect to INS gene expression (Fig. 3e). Approximately 70% of transfected cells were INS**^+^** beta-like-cells, while roughly 30% were INS**^−^**, non-beta-like-cells (possibly of mixed cell phenotypes). This result might be due to unanticipated differentiation of the cells into different phenotypic classes during culture or could possibly indicate a non-clonal nature of the reference HIT-T15 cell line we obtained from ATCC. It was recently reported that pancreatic beta-cell identity is maintained by DNA methylation-mediated repression of Arx [21], [22]. Deletion of DNA methyl-transferase gene in INS**^+^** beta-cells converts them into GCG**^+^** alpha-cells by derepression of Arx transcription repressor in beta-cells [39], [40]. The red population of cells showed a low level of INS and Pdx1 transcript expression and a high level of GCG and Arx transcript expression (Fig. 4), suggesting conversion of beta-cells into alpha-cells, likely by over-expression of Arx through lack of DNA methylation-mediated repression. Regardless, our results argue that care must be taken in interpretation of previous publications on gene expression and functional profiling of HIT-T15 cells [41], [42], [43] with respect to identification of genes and properties as being exclusive correlates of authentic beta-like phenotypes. Some of the previously reported findings may instead be confounded by the significant population of non-beta-cells.

Our fluorescent reporter enables rapid identification and FACS purification of a small percentage of unambiguously identified INS^+^ beta-like-cells in a mixed population with a significant proportion of non-beta-like-cells (Fig. 4b). We found that GFP^+^ cells expressed insulin transcript, while mCherry^+^ cells expressed glucagon (Fig. 4b & c). Furthermore, in comparison to mCherry^+^ cells, GFP^+^ cells produced significantly larger inward voltage-gated calcium currents, glucose-stimulated elevation of [Ca^2+^]_i_, and larger voltage-stimulated secretion (Fig. 5 and Fig. 6). These characteristics are all indicative of authentic beta-cells [23], [44]. In contrast, mCherry^+^ cells exhibited greater variability in the amplitude of calcium currents and exocytotic responses, perhaps indicating a mixture of different cellular phenotypes in this population. In the future, we will use our reporter to isolate purely homogeneous beta- or beta-like-cells, which will later be used for more accurate genetic, epigenetic, and functional profiling. Our approach should also allow us to compare gene expression and functional phenotypes of beta- or beta-like-cells with other types of pancreatic cells that may be present in the mCherry^+^ class of cells (α, δ, and PP cells, or perhaps even exocrine acinar cells).

Human islet transplantation using the Edmonton protocol is an effective treatment for type I diabetes [26]. Its widespread applicability, however, is limited because of a scarcity of donor tissue. Properly and correctly differentiated beta-cells from human pluripotent stem cells could potentially overcome this limitation. Most current human pluripotent stem cell differentiation protocols [45], [46] have limited reproducibility, low yield of beta-like-cells, and most importantly, the absence of glucose responsiveness [45]. We expect that our reporter will facilitate easy and quick evaluation of new differentiation protocols designed to produce clinically useful beta-cells—it could be stably introduced into human pluripotent stem cells [47] or transduced into different stage cells using an adenoviral vector [48], [49]. In addition, the reporter system may be used as a high-throughput screening for reprogramming non-insulin-producing cells to insulin-producing cells. Further additions and modifications to our approach could be easily incorporated, depending on the particular phenotypic property desired. For example, other promoters could be used to mark different cell- or tissue-specific lineages and to further test their homo/heterogeneity.

## Materials and Methods

### Cell culture, transfection, FACS sorting and q-RT-PCR

HIT-T15 [6] (ATCC, USA), INS-1 823/13 (a gift from Chris Newgard [19]) and NT-2 cells [50] were cultured as descried previously. Human dermal fibroblasts (ScienCell) were cultured in MEM alpha medium (Invitrogen) with 0.1 mM MEM nonessential amino acid, 10% FBS, and penicillin/streptomycin. MDA-MB-231 cells were cultured in DMEM medium with 10% FBS and penicillin/streptomycin. All cells were incubated at 37°C with 5% CO_2_ in a humidified incubator. For all transfection experiments, unless otherwise specified, we used Lipofectamine 2000 (Invitrogen) using the manufacturer's instructions. For making stable HIT-T15 cells, cells were transiently transfected with our reporter (pA-Cre-pINS-pCMV-loxP-mCherry-pA-loxP-EGFP-pA). Two days after transfection, cells were split at a 1∶10 ratio with fresh growth medium into 10 cm tissue culture dishes and selected with 800 µg/ml G418 until colonies were visible. The whole populations of stable transfectants or individual colonies were screened for expression of fluorescent proteins.

Expression of fluorescent proteins was examined with an Olympus IX70 fluorescence microscope (Olympus, Japan) or a Leica TCS SP5 confocal microscope (Leica Microsystems, Germany). Images were analyzed and edited in MetaMorph software.

For cell sorting, cells were trypsinized after imaging, suspended in medium, and subjected to FACS sorting for GFP^+^ and mCherry^+^ cells. FACS was performed by the USC FACS Core Facility, using a BD FACSAria cell sorter. After cell sorting, total RNAs were isolated from GFP^+^ or mCherry^+^ cells using Trizol (Invitrogen). After digestion of total RNAs with Turbo DNase (Ambion), cDNAs were made from the total RNAs with an iScript cDNA synthesis kit (Bio-Rad), according to the manufacturer's instructions. The cDNA was diluted three-fold with water prior to quantitative PCR (q-PCR) analysis. Gene-specific q-PCR primer/probe sets (customized TaqMan Gene Expression Assays, Applied Biosystems) for hamster INS, GCG, GAPDH, Cyclophillin, and human Pdx-1 and equivalent amounts of cDNA generated as a template were used for q-PCR. Reactions were performed in for each sample using TaqMan Universal PCR Master Mix with a CFX-96 system (Bio-Rad). The q-PCR was performed using the manufacturer's instructions. For each sample, expression of marker genes was normalized to GAPDH or Cyclophillin. Data are expressed as a relative expression level. For detection of Arx that is not well studied in the hamster system, we performed q-PCR using mouse primers (F:5′-TTCCAGAAGACGCACTACCC; R: 5′-TCTGTCAGGTCCAGCCTCAT [21]) as descried previously [50].

### Immonofluorescent staining and confocal microscopy

Cells grown on sterilized, 0.4% gelatin-coated patch-clamp dishes were washed with PBS and fixed with 4% paraformaldehyde for 20 minutes at room temperature. For detection of Cre nuclear proteins, dishes were permeabilized for 10 minutes with 0.25% Nonidet P-40 (NP40). Cells were blocked overnight at 4°C with 5% fetal goat serum, 1% bovine serum albumin, and 0.1% Triton X-100, and incubated for 1 hour with the primary mouse monoclonal anti-Cre antibody (Sigma-Aldrich, a gift from Le Ma) diluted in blocking solution (1∶500). Cells were then washed and incubated for 1 hour with the secondary anti-mouse Cy-2 or Cy-3 conjugated antibody (1∶250; Jackson ImmunoResearch, a gift from Le Ma). Images were taken using a Leica TCS SP5 confocal microscope. EGFP and mCherry were visualized by endogenous fluorescence.

Fluorescent signal of EGFP and mCherry in yellow cells were visualized by a Leica TCS SP2 AOBS confocal microscope system (Leica-Microsystems, Heidelberg, Germany) using 10× and 63× magnifications. A Leica DM IRE2 inverted microscope was powered by Argon and HeNe lasers for the detection of EGFP (excitation at 488 nm, emission at 495–550 nm) and mCherry (excitation at 594 nm, emission at 600–750 nm) fluorescence. Images were collected in xyz series and analyzed by Leica LCS imaging software (LCS 3D, Process, and Quantify packages).

### DNA constructs and expression

All constructs were made using standard molecular cloning methods. To construct phINS-EGFP-pA-pCMV-DsRed2-pA, plasmid phINS-EGFP [6] was digested using *SalI* and blunted at both sites with Klenow DNA polymerase (NEB), obtaining phINS-EGFP-pA fragment. The fragment was then subcloned upstream of CMV promoter in pDsRed2-N1 (Clontech) digested using *AseI* and blunted at both sites with Klenow.

For constructing pCMV-L-mCherry-pA-L-EGFP-pA, a fragment of L-mCherry-pA-L containing *HindIII* and *XhoI* restriction enzyme sites at each 5′ and 3′ ends, respectively, was obtained by polymerase chain reaction (PCR) using a template (lox2272-mCherry-loxP in pBlue-script SK, a gift from Li Zhang [51]), and forward (5′-catggg**aagctt** ataacttcgtatagcatacattatacgaagttatGCCACC**ATG**GTGAGCAAGGG- CGAGGA) and reverse (5′-ggaattc**ctcgag** ataacttcgtataatgtatgctatacgaagttatAGATC-TGTCGAGGCCGCGAATTAAAAAACC) primers. Underlined sequences are the LoxP site and its reverse complement, and the bold capitalized **ATG** is the start codon of mCherry. The bold fonts are the restriction enzyme sites for digestion. The PCR product was cut by *HindIII* plus *XhoI* and blunted at the *XhoI* site with Klenow. The *HindIII-XhoI* (blunt) fragment was inserted into pEGFP-N1 (Clontech) digested with *HindIII* plus *SalI* and blunted at *SalI* site with Klenow.

To obtain plasmid phINS-Cre-pA, a Cre-pA fragment was generated by PCR using pCMV-Cre-pA (a gift from Li Zhang) as a template, and forward: 5′-ggaattc- **ccgcgg ATG**CCCAAGAAGAAGAGGAAGGTGTC, reverse (5′-gaattc**cttaag**gagctc GAACAAACGACCCAACACCCGTG) primers. After digesting with *SacII* and *AflII*, the PCR product was inserted downstream of human INS promoter in phINS-EGFP that was digested with *SacII* and *AflII* and removed EGFP-pA by gel extraction, generating plasmid phINS-Cre-pA. To combine phINS-Cre-pA with pCMV-L-mCherry-pA-L-EGFP-pA, phINS-Cre-pA fragment was obtained from plasmid phINS-Cre-pA by digesting with *SalI* plus *AflII* and blunted at both sites with Klenow. The fragment was inserted in the reverse orientation (overwhelmingly, but not in the right orientation) upstream of CMV promoter in pCMV-L-mCherry-pA-L-EGFP-pA digested with *AseI* and blunted at both sites with Klenow. DNA sequences were confirmed using standard sequencing protocols (USC DNA Sequencing Core Facility).

### Electrophysiology of HIT T15 cells

Cells were patch-clamped 2–4 days after plating. Current recordings were obtained in conventional whole cell patch-clamp configuration with an Olympus IX70 inverted microscope, EPC-9 amplifier, and Pulse software (HEKA Electronics). The glass-bottomed chambers with adherent cells were washed with PBS and then filled with standard extracellular solution consisting of 140 mM NaCl, 2.8 mM KCl, 10 mM HEPES, 1 mM MgCl_2_, 2 mM CaCl_2_, 10 mM glucose, pH adjusted to 7.2–7.4, and osmolarity adjusted to 300–310. The pipette solutions contained 10 mM NaCl, 145 mM cesium glutamate, 10 mM HEPES, 2 mM CaCl_2_, 1 mM MgCl_2_, 0.1 mM EGTA, 2 mM ATP, 0.3 mM GTP, titrated with 5 M CsOH to pH 7.2–7.4, and osmolarity adjusted to 290–300.

### Measurement of [Ca^2+^]_i_ in response to glucose stimulation

In order to observe calcium changes associated with glucose stimulation, we used the ratiometric calcium dye Fura2-AM (Invitrogen). The intracellular Ca^2+^ concentration was determined by the ratio of the excitation of the ratiometric calcium dye Fura2-AM (Invitrogen) at 350 and 380 nm using the Polychrom V by Till Photonics. Emission was measured at 500–520 nm using a Photometric Cascade CCD camera and Metafluor Software.

Cells were incubated at room temperature for 1 hour in standard patch-clamp external solution with a modified glucose concentration of 3 mM and supplemented with 4 µM Fura2-AM. The cells were washed three times with 3 mM glucose external solution before recording. Fura-2 fluorescence was detected with a timelapse of 300 ms at 500–520 nm wavelength following excitation at 350 nm (F350) and 380 nm (F380). The ratio, F350/F380, was calculated after subtracting background from each wavelength using Igor programming software. After recording fluorescence measurements in 3 mM glucose, high-glucose extracellular solution was added for a final concentration of 14 mM glucose to stimulate glucose response. The ratio, F350/F380, was again obtained using Igor software.

## Supporting Information

Figure S1 **Transfection efficiency in MB-231 and HIT-T15 cells by pEGFP-N1.**
(DOC)

Figure S2 **Transiently transfected HIT-T15 and beta-TC-6 cells with the indirect dual-color reporter.** (a, b) Confocal images of HIT-T15 (a) and beta-TC-6 (b) cells. Green and red fluorescence is colocalized in one cell.
(DOC)
