# Supplementary material for: A Novel Dual-Color Reporter for Identifying Insulin-Producing Beta- Cells and Classifying Heterogeneity of Insulinoma Cell Lines
Source: PLoS One. 2012 Apr 18;7(4):e35521. doi: 10.1371/journal.pone.0035521 (PMC3329476; doi:10.1371/journal.pone.0035521)

### Supporting Information 1

### When transiently transfected with pEGFP-N1, ~70% of cells showed GFP expression for both MB-231 and HIT-T15 cell lines, indicating similar transfection efficiencies (Fig. S1).


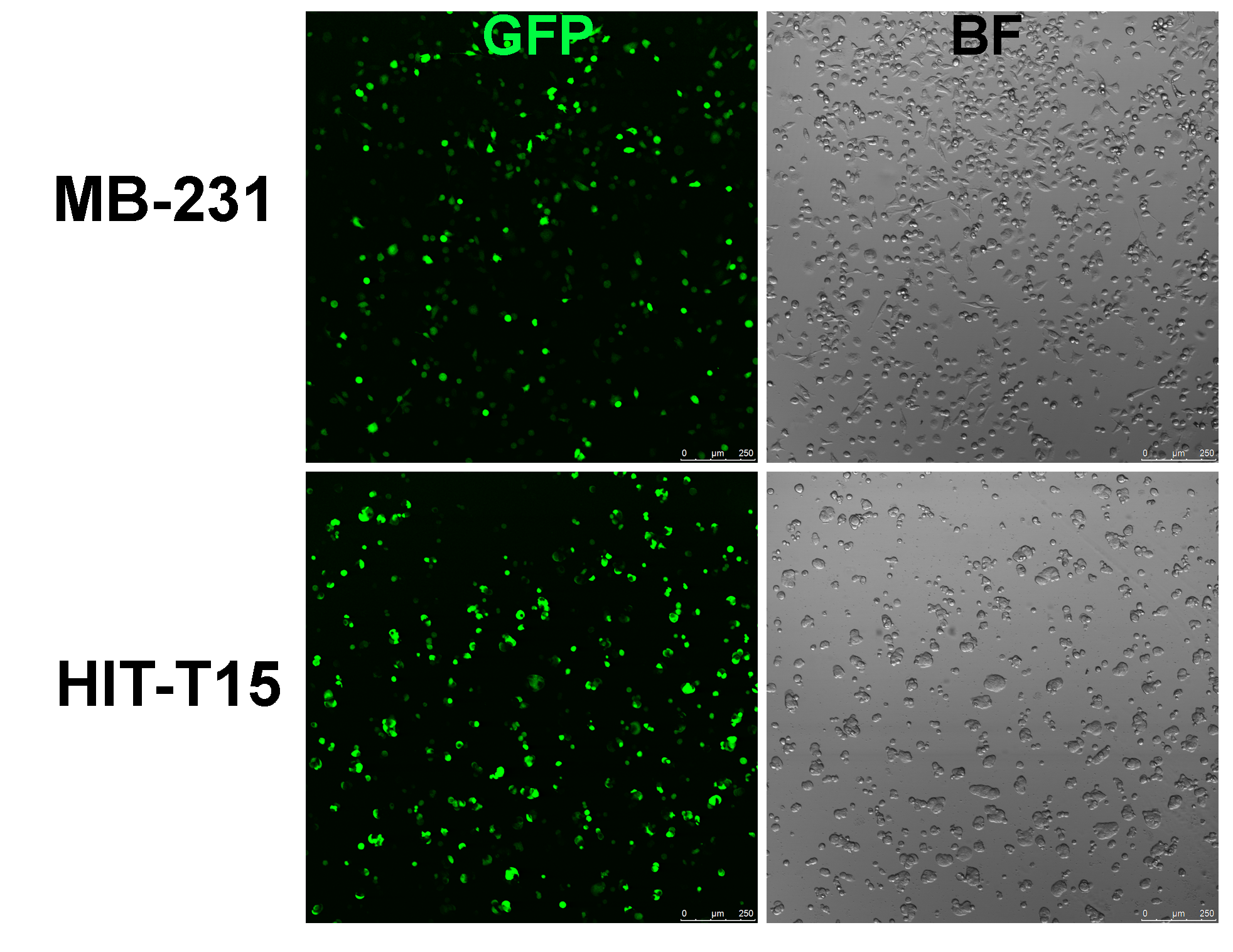

Supplement: Figure S1 — Transfection efficiency in MB-231 and HIT-T15 cells by pEGFP-N1. (DOC) [file pone.0035521.s001.doc]
