# Supplementary material for: A Novel Dual-Color Reporter for Identifying Insulin-Producing Beta- Cells and Classifying Heterogeneity of Insulinoma Cell Lines
Source: PLoS One. 2012 Apr 18;7(4):e35521. doi: 10.1371/journal.pone.0035521 (PMC3329476; doi:10.1371/journal.pone.0035521)

### Supporting Information 2

Transiently transfected HIT-T15 cells with the indirect dual-color reporter showed yellow fluorescence. We imaged the cells with confocal microscopy to confirm that the yellow cells showed both green and red fluorescence, rather than being two separate cells stacked atop one another (Fig. S2a). We also transiently transfected beta-TC-6 cells (mouse insulinoma cell line) with the reporter and observed that they also showed both green and red (yellow) fluorescence (Fig. S2b).

Beta-TC-6 cells (a gift from Bangyan Stiles, USC) were cultured in DMEM medium with 20% FBS and penicillin/streptomycin.


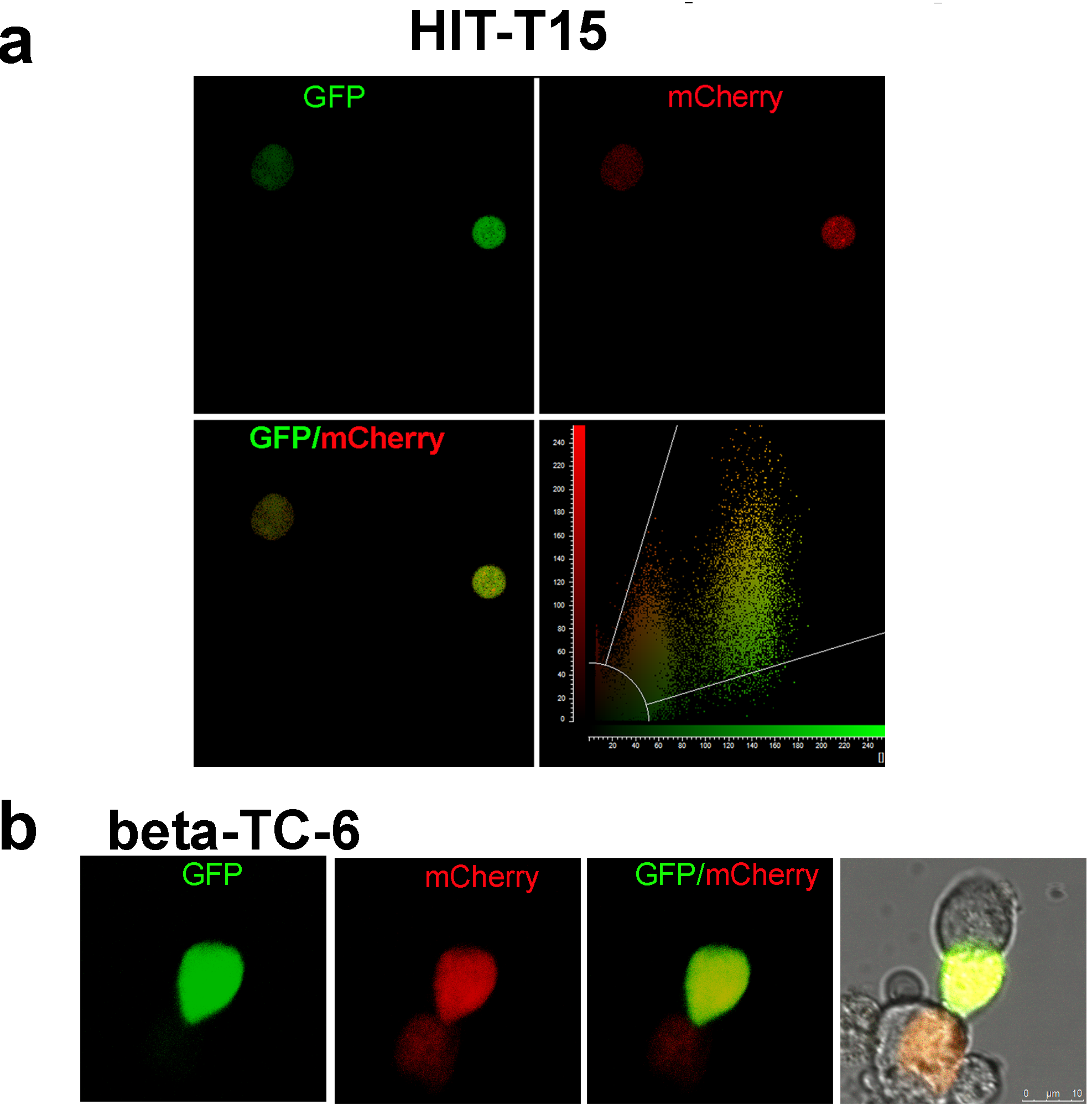

Supplement: Figure S2 — Transiently transfected HIT-T15 and beta-TC-6 cells with the indirect dual-color reporter. (a, b) Confocal images of HIT-T15 (a) and beta-TC-6 (b) cells. Green and red fluorescence is colocalized in one cell. (DOC) [file pone.0035521.s002.doc]
